# Molecular Detection of *Rickettsia* spp. and *Coxiella burnetii* in Cattle, Water Buffalo, and *Rhipicephalus* (*Boophilus*) *microplus* Ticks in Luzon Island of the Philippines

**DOI:** 10.3390/tropicalmed5020054

**Published:** 2020-04-04

**Authors:** Remil L. Galay, Melbourne R. Talactac, Bea V. Ambita-Salem, Dawn Maureen M. Chu, Lali Marie O. dela Costa, Cinnamon Mae A. Salangsang, Darwin Kyle B. Caracas, Florante H. Generoso, Jonathan A. Babelonia, Joeneil L. Vergano, Lena C. Berana, Kristina Andrea C. Sandalo, Billy P. Divina, Cherry R. Alvarez, Emmanuel R. Mago, Masako Andoh, Tetsuya Tanaka

**Affiliations:** 1 Department of Veterinary Paraclinical Sciences, College of Veterinary Medicine, University of the Philippines Los Baños, College, Laguna 4031, Philippines; Beaambita@gmail.com (B.V.A.-S.); dmchu@up.edu.ph (D.M.M.C.); lodelacosta@up.edu.ph (L.M.O.d.C.); casalangsang@up.edu.ph (C.M.A.S.); dbcaracas@up.edu.ph (D.K.B.C.); kacsandalo@gmail.com (K.A.C.S.); bpdivina@up.edu.ph (B.P.D.); 2 Department of Clinical and Population Health, College of Veterinary Medicine and Biomedical Sciences, Cavite State University, Indang, Cavite 4122, Philippines; melbourne.talactac@gmail.com (M.R.T.); florantegeneroso@gmail.com (F.H.G.); jonathanbabelonia@gmail.com (J.A.B.); joeneilbergs@gmail.com (J.L.V.); lenaberana777@gmail.com (L.C.B.); cherryreyesalvarez@gmail.com (C.R.A.); emman.mago.7081@gmail.com (E.R.M.); 3 Laboratory of Public Health, Joint Faculty of Veterinary Medicine, Kagoshima University, Korimoto 1-21-24, Kagoshima 890-0065, Japan; masako@vet.kagoshima-u.ac.jp; 4 Laboratory of Infectious Diseases, Joint Faculty of Veterinary Medicine, Kagoshima University, Korimoto 1-21-24, Kagoshima 890-0065, Japan

**Keywords:** *Coxiella burnetii*, Rickettsia, Q fever, rickettsiosis, tick-borne pathogens

## Abstract

*Rickettsia* and *Coxiella burnetii* are zoonotic, tick-borne pathogens that can cause febrile illnesses with or without other symptoms in humans, but may cause subclinical infections in animals. There are only a few reports on the occurrence of these pathogens in cattle and water buffalo in Southeast Asia, including the Philippines. In this study, molecular detection of *Rickettsia* and *C. burnetii* in the blood and in the *Rhipicephalus* (*Boophilus*) *microplus* ticks of cattle and water buffalo from five provinces in Luzon Island of the Philippines was done. A total of 620 blood samples of cattle and water buffalo and 206 tick samples were collected and subjected to DNA extraction. After successful amplification of control genes, nested PCR was performed to detect *gltA* of *Rickettsia* and *com1* of *C. burnetii*. No samples were positive for *Rickettsia*, while 10 (cattle = 7, water buffaloes = 3), or 1.6% of blood, and five, or 1.8% of tick samples, were *C. burnetii*-positive. Sequence analysis of the positive amplicons showed 99–100% similarity to reported *C. burnetii* isolates. This molecular evidence on the occurrence of *C. burnetii* in Philippine ruminants and cattle ticks and its zoonotic nature should prompt further investigation and surveillance to facilitate its effective control.

## 1. Introduction

In the Philippines, cattle and water buffalo are of economic importance, contributing significantly to the agricultural sector. They are mainly utilized in the production of meat, milk, and additionally for water buffalo, for draft power [1]. The total population of cattle and water buffalo (locally known as carabao in the Philippines) as of January 2020 is 2.54 M and 2.87 M, respectively [2,3]. Through efforts of the Philippine government, there has been a steady increase in large ruminant production in recent years. Diseases that can affect the health and production of these animals, including those that are zoonotic, have an impact on the lives of animal raisers and product consumers. Tick-borne pathogens (TBPs) affect the ruminant population worldwide and are widely distributed, particularly in the tropics and subtropics, representing an essential proportion of all animal diseases that constrain the livelihood of farmers. Among these TBPs of ruminants, *Rickettsia* and *Coxiella burnetii* can pose great threats to public health. However, little is known about the occurrence of these TBPs in Southeast Asia, including the Philippines.

*Rickettsia* and *C. burnetii* are obligate, intracellular, Gram-negative bacteria that can cause febrile illnesses of varying severity in humans. Aside from being transmitted by ticks, *Rickettsia* can also be transmitted by fleas, lice, and mites [4], whereas *C. burnetii* is usually acquired by humans through the inhalation of contaminated aerosol and ingestion of contaminated milk from infected animals [5]. Rickettsioses are known to occur worldwide, and have historically shown different patterns of emergence, from seasonal outbreaks to large-scale epidemics [6]. They are endemic in North and South America, Europe, Africa, and Asia [7]. Meanwhile, *C. burnetii* has been detected almost all over the world, except New Zealand [8,9]. Both pathogens can infect a wide range of hosts, including wild and domestic animals, as well as humans. Serological [10,11,12,13,14,15] and molecular studies [16,17,18,19,20,21,22] have demonstrated the occurrence of these pathogens in ruminants and associated ticks, implying their role as a potential source of infection for livestock workers. 

*Rickettsia* is mainly classified into the spotted fever group (SFG) and the typhus group (TG), based on serological characteristics, with the former further divided into the ancestral group (AG) and the transitional group (TRG) [23]. The clinical presentation of rickettsioses in animals vary from subclinical to severe. In cattle and goats, although seroconversion was observed upon inoculation; clinical signs, however, were not observed [24]. In contrast, infections with *Rickettsia* are considered the second highest cause of non-malarial febrile illness in humans worldwide, and are associated with high morbidity and rising mortality rates [25].

Infection with *C. burnetii* in animals is commonly asymptomatic, but can also lead to abortion and stillbirths in pregnant mammals [5]. It can also induce pneumonia and the delivery of weak calves. The female uterus and mammary glands are the primary sites of chronic *C. burnetii* infection [26]. In cattle, metritis is frequently the only manifestation of the disease [27]. Domestic ruminants represent the most frequent source of human infection, of which cattle, goats, and sheep are considered as the primary reservoirs [26,28]. In humans, *C. burnetii* infection, known as Q fever, may be acute or chronic [5]. The acute stage may be asymptomatic, or it is exhibited by non-specific clinical signs, such as fever, headache, nausea, vomiting, and myalgia. The chronic stage is characterized by more severe pathology, such as endocarditis, hepatitis, vasculitis, and chronic fever states [29].

The cattle tick *Rhipicephalus* (*Boophilus*) *microplus* is widely spread in the Philippines. Several studies have been done on TBPs affecting cattle [30]. However, few studies have been done to determine the occurrence of *Rickettsia* and *C. burnetii.* Camer et al. [31] demonstrated the presence of antibodies against the SFG and TG *Rickettsia* and *C. burnetii* in humans by indirect immunofluorescent antibody test (IFAT), whereas Cardona [32] used complement fixation for serological testing of *C. burnetii* in domestic ruminants and humans. Recently, a study from our group utilizing nested PCR provided evidence for the presence of *Rickettsia* in dogs in the Philippines [33]. Here, we investigate the presence of *Rickettsia* and *C. burnetii* in cattle and water buffalo, as well as *R.* (*B.*) *microplus* ticks, in selected provinces in Luzon, Philippines, through nested PCR. Knowledge of the presence of these pathogens is crucial in ascertaining the potential risk for people working closely with those animals, and in implementing appropriate control measures.

## 2. Materials and Methods 

### 2.1. Study Area and Collection of Samples

**Figure 1:**
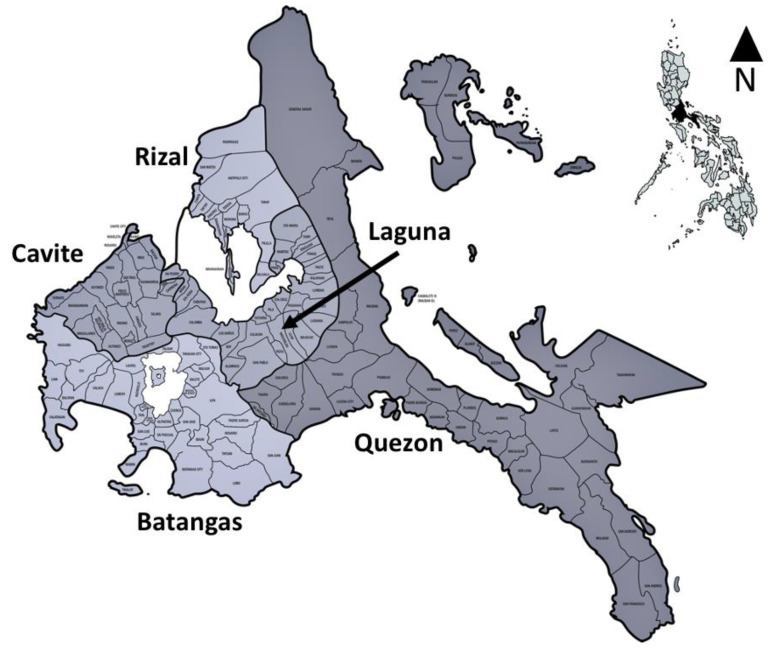
Map of the study area, Region IV-A or CALABARZON, comprised of five provinces (Cavite, Laguna, Batangas, Rizal, and Quezon). The smaller map was created using the online software MapChart (https://mapchart.net/).

Blood of cattle and water buffalo, as well as ticks, were collected in five provinces in Luzon, Philippines—namely, Cavite, Laguna, Batangas, Rizal, and Quezon, collectively known as CALABARZON or Region IV-A (Figure 1). This region, located at 14.1008° N, 121.0794° E, is among the regions of the Philippines with a high population of large ruminants, having 269,677 cattle [2] and 177,661 water buffaloes [3]. The collection of samples was done between March 2016 and October 2019, from a total of 620 animals (512 cattle and 108 water buffaloes), regardless of breed type, age and sex, and health status, from selected commercial and backyard raisers who agreed to participate in this study. Information on the animals, including health status, was noted. Blood was collected from each animal using a 5 mL syringe with an 18G needle, and was transferred to a tube containing ethylenediaminetetraacetic acid (EDTA). Aside from blood, ticks were also collected from those animals, if present, using a specialized tick removal tool (O’tom / Tick Twister, H3D, Lavancia, France) and were placed in glass vials. All ticks were morphologically identified as *R.* (*B.*) *microplus* in the laboratory under a stereomicroscope, based on the description of Barker and Walker [34], and were sorted according to developmental stage and sex. Nymphs and male adults collected from the same animal were pooled separately and then processed accordingly, while partially-fed and engorged female ticks were processed individually. The collection procedures in the animals have been approved by the Institutional Animal Care and Use Committee (IACUC) of the College of Veterinary Medicine, University of the Philippines Los Baños, and of Cavite State University, following applicable national guidelines.

### 2.2. Extraction of DNA from Blood and Tick Samples

Commercial extraction kits (innuPREP DNA/RNA Mini Kit and blackPREP Tick DNA/RNA Kit, Analytik Jena, Jena Germany) were used to extract DNA from blood and tick samples following the manufacturer’s protocol, with some modifications. Specifically, the modifications included tick homogenization in lysis buffer, with the aid of a digital cell disruptor (Disruptor Genie^®^, Scientific Industries Inc., New York, United States), and were incubated at room temperature for at least 30 min to allow complete lysis. All DNA samples were stored at −40°C until used for PCR assays.

### 2.3. PCR Detection of Control Genes and Pathogens

To confirm the success of DNA extraction before pathogen detection, conventional PCRs for the amplification of *actin* and *mt-rrs* genes in the blood and tick DNA samples, respectively, were performed, as described previously [35,36]. After the successful amplification of control genes, nested PCRs targeting the citrate synthase (*gltA*) gene of *Rickettsia* [37] and the *com1* gene, which encodes a 27-kDa outer membrane protein of *C. burnetii* [38], were performed. PCR mixtures consisted of 2x PCR buffer, 10 pmol each of forward and reverse primers, polymerase (Tks Gflex DNA Polymerase, TaKaRa, Shiga, Japan), nuclease-free water, and a template (1 µL DNA or first PCR product for 10 µL mixtures). All primers used in this study are listed in Table S1 in supplementary materials, while the PCR conditions are shown in Table S2 in supplementary materials. Negative controls containing nuclease-free water and positive controls containing *R. japonica* and *C. burnetii* DNA were included. Electrophoresis of PCR products was done in 2% agarose gel in 1x Tris-acetate-EDTA (TAE) buffer, and bands were visualized through a gel documentation system (Bio-Print, Vilber, Lourmat, France) after being stained with ethidium bromide in 1x TAE.

### 2.4. Sequence and Data Analysis

Upon visualization of positive bands, amplicons were excised and purified using a commercial kit (NucleoSpin Gel and PCR Clean-up kit, Macherey-Nagel, Leicestershire, England) following the recommended protocol. The purified amplicons were sent to a third-party laboratory for capillary sequencing, using the forward primer for nested PCR. The similarity of obtained amplicon sequences was determined by multiple nucleotide sequence alignment using an online software MAFFT version 7 (https://mafft.cbrc.jp/alignment/server/index.html). The nucleotide sequence readings obtained were compared to previously reported sequences using the Basic Local Alignment Search Tool, or BLAST (https://blast.ncbi.nlm.nih.gov/Blast.cgi). A phylogenetic tree was constructed using online software (http://www.phylogeny.fr). The detection rate of the pathogen was determined by dividing the number of positive samples by the number of samples per source (e.g. cattle, water buffalo, and ticks) and is expressed as a percentage.

## 3. Results

Table 1 shows the breakdown of blood and tick samples collected from the five provinces in Luzon, Philippines, and the corresponding results of the nested PCR assays. The 620 animals were comprised of 108 males and 512 females. With regard to their purpose, 271 animals are being raised for milk (dairy type), 285 for meat (beef type), and 64 as draft animals. Only 165 animals were found to have ticks at the time of sample collection. A total of 206 tick samples, comprised of 14 pools of nymphs, 31 pools of male ticks, and 161 individual female ticks, was tested.

DNA was successfully extracted from each sample, as shown by positive amplification of the control genes *actin* and *mt-rrs.* In the nested PCR assay, bands of 381 bp and 438 bp were considered positive for *Rickettsia* and *C. burnetti*, respectively, as observed in respective positive controls. All blood and tick samples from both animal hosts were negative for *Rickettsia*. In contrast, there were 10 blood samples positive for *C. burnetii*, of which seven (1.4%) were from cattle and three (2.8%) were from water buffalo (Table 1). The positive animals were all female, and came from seven different municipalities in two provinces (Rizal and Quezon). Additionally, six of those animals are being raised for dairy, three for meat, and one as a draft animal. Furthermore, five (2.3%) tick samples were also positive for *C. burnetii.* Three of those tick samples were females, and two were pooled male tick samples, which were all collected from Quezon province.

All *C. burnetii* positive amplicons were subjected to nucleotide sequencing. The alignment of obtained nucleotide sequences revealed that all the amplicons are 100% similar. Furthermore, BLAST analysis revealed that the amplicons share 100% identity with reported *C. burnetii* isolates, such as the RSA439, CPBBU1, and Fars-GH4 strains, having 99% query coverage. Construction of a phylogenetic tree was attempted. However, the isolates from this study and those isolated in other countries were grouped together, with no observed ramifications (data not shown). The sequence of one amplicon was deposited in the DNA Data Bank of Japan (accession number: LC534651). 

## 4. Discussion

The present study was conducted to determine the presence of two zoonotic, tick-borne pathogens, *Rickettsia* and *C. burnetii,* in cattle, water buffalo, and ticks in the Philippines. None of the samples tested positive for *Rickettsia,* suggesting the absence of the pathogen in the areas where the samples were collected. Prior to this study, there were no reports of the occurrence of *Rickettsia* in these animal hosts in the Philippines. Previous studies in the country only reported the detection of antibodies against SFG *Rickettsia* in dogs and rats [39], as well as antibodies against SFG and TG *Rickettsia* in humans [31]. Recently, our group detected *Rickettsia* in dogs from Laguna, one of the provinces in this study, through the same nested PCR employed in this study. It was found that the amplicons were highly identical to *R. japonica* after sequence analysis [33]. 

Evidence of rickettsiosis in ruminants have been reported in other countries. Serological studies using ELISA and IFAT have been mostly successful in demonstrating the presence of antibodies against *Rickettsia* in ruminants, including cattle [15,40,41,42]. On the other hand, previous studies on PCR detection in the blood reported negative results [40,41], which is due to the low titer or absence of rickettsemia [41,43]. The same reason may explain the negative results obtained in this study. A study on the detection of vector-borne pathogens, including *Rickettsia* in ungulates in Hungary, was conducted using real-time PCR targeting *23S rRNA* and *gltA* for *R. helvetica* and other *Rickettsia*, respectively [44]. Whereas none of the blood samples from the water buffaloes tested positive for *R. helvetica* or other rickettsiae, *R. helvetica* was detected in a blood sample and a spleen sample from a deer. Moreover, a deer spleen sample also tested positive for an unknown *Rickettsia* [44]. A recent study in Cameroon showed the successful detection of *Rickettsia* in cattle using conventional PCR targeting the *16s rDNA* gene, with a prevalence of 14.3% [45].

Nevertheless, aside from the possible reasons for the negative results discussed above, the limitations of the detection method employed in this study must also be considered. Whereas nested PCR is known for high sensitivity, it may be unable to detect *Rickettsia* if the bacterial load in the blood is less than 100/mL, and if the DNA yield after extraction is poor [46]. The gene targeted for *Rickettsia* detection in this study is *gltA,* which is considered a highly conserved gene and is very useful for phylogenetic analysis [47]. However, for detection, it is most useful for SFG and TG rickettsiae. For future studies, a real-time PCR targeting a 74 bp fragment of *gltA* should be performed due to its high sensitivity, as it is capable of detecting one copy number per reaction [48]. Moreover, sequential assays targeting other genes, such as *ompA* and *ompB,* in addition to *gltA,* may be performed to ensure higher sensitivity [49].

Concerning the negative results of *Rickettsia* nested PCR in ticks in the current study, they are in contrast to the report of previous studies that were able to detect *Rickettsia* in various ticks from domestic ruminants, with *Rickettsia* being the most detected tick-borne pathogen [50,51,52,53]. In those studies, the *gltA* gene was amplified through real-time PCR or nested PCR. A study in Thailand utilizing conventional PCR targeting *gltA* also reported the detection of *Rickettsia* in *R. (B.) microplus* ticks [21]. Aside from pathogenic species, endosymbiont *Rickettsia* has also been identified and characterized in *Ixodes* ticks [54,55]. Future studies should also employ other methods and target different genes for the detection of *Rickettsia* in ticks, as mentioned above. 

The current findings on the occurrence of *C. burnetii* in large ruminants and cattle ticks support a previous report on the detection of antibodies against *C. burnetii* in cattle and water buffalo in the country, using a complement fixation test [32]. The origin of the seropositive animals in that study was different from the origin of the nested PCR positive animals in our study, which together provide evidence that *C. burnetii* is present in several provinces of the Philippines. BLAST analysis revealed a very high identity shared with reported isolates of *C. burnetii* from other countries. Due to the highly conserved nature of the targeted *com1* gene [38], we were unable to come up with a good phylogenetic analysis. Hence, it is recommended that another gene, such as *16s rRNA*, be amplified to further validate the amplicons and to elucidate the relationship of *C. burnetti* isolates from the Philippines with those from other countries.

The *C. burnetii-*positive animals in this study were not observed with any clinical signs at the time of blood collection, except for two that were noted to have poor body condition score, suggesting possible subclinical infection. This observation corroborates a previous report that seropositive cattle may be asymptomatic [42]. The positive blood samples in this study were from female animals, and there was no mention by the animal raisers during sample collection whether those animals had a history of any reproductive problems. Coxiellosis can cause reproductive problems, such as metritis [27], abortion, delivery of premature offspring, stillbirth, and weak offspring (or APSW complex, as termed by Agerholm [56]) in animals. On another note, subclinically infected animals identified in this study present a greater risk to the people working with them, because they can shed *C. burnetii* through their feces, vaginal fluids, milk, and parturition byproducts [9,57,58,59,60] without being identified as infected, due to absence of clinical signs. Moreover, six of those *C. burnetii*-positive animals are being raised for milk production, hence posing a health risk if the milk from those animals is consumed by humans without being pasteurized.

*Coxiella burnetii* was also found to be present in *R. (B.) microplus* ticks collected in this study. This result supports the previous report of Muramatsu et al. [61], wherein *C. burnetii* was also detected in two engorged *R. (B.) microplus* female ticks collected in Thailand through RFLP-nested PCR that also targeted the *com1* gene. In contrast, another study in Thailand reported the non-detection of the pathogen in that tick after conventional PCR targeting the *16s rRNA* gene [21]. To date, there is still no report proving the role of *R. (B.) microplus* in transmitting *C. burnetii* in cattle. However, there is a possibility that this tick can harbor the pathogen, since a related *Coxiella*-endosymbiont has been found in different developmental stages and organs of the tick [62]. None of the animals from which the *C. burnetii* positive ticks were collected showed a positive result in the blood samples, which may be due to the absence or very low bacteremia at that time.

Serological evidence for rickettsiosis and Q fever in humans has already been reported in the Philippines [31,32]. In the study of Camer et al., [31] antibodies against *Rickettsia* had been detected in febrile patients in two hospitals in the country. However, the authors did not detect antibodies against *C. burnetii*. On the other hand, a more recently published study by Cardona [32] demonstrated the presence of antibodies against *C. burnetii* in humans from two localities. Evidence of Q fever in humans has also been reported in neighbouring Asian countries, such as Thailand [63] and Malaysia [64]. In Thailand, seropositive subjects included people working with ruminants, which led to the conclusion that exposure to those animals presents the risk of acquiring infection [63]. The findings in this study suggest that humans working closely with *C. burnetii-*positive animals should also be tested.

In summary, nested PCR showed that *Rickettsia* is absent in all blood and tick samples tested, whereas *C. burnetii* was found in cattle and water buffalo from two provinces and ticks in one province. To our knowledge, this study provides the first molecular evidence that *C. burnetii* is present in animal and tick populations in the Philippines. This result necessitates more thorough studies on prevalence, geographical distribution, transmission dynamics (including in other animals), and risk assessment in those two provinces, as well as in other regions of the Philippines. Serological studies should be done involving the people working closely with cattle, water buffalo, and other ruminants, to further assess exposure and risk of spreading. Molecular assays targeting other genes of *Rickettsia* and *C. burnetti* should be performed to improve detection sensitivity and further characterize positive samples. Lastly, a “One Health” approach involving medical and environmental professionals should be applied to control this threat to public health. 

## Figures and Tables

**Table 1:** Number and percent (%) of blood samples from cattle, water buffalo, and ticks collected from selected provinces in Luzon, Philippines, that tested positive for *Coxiella burnetii*, based on nested PCR. All the samples were negative for *Rickettsia*. *n* = number of examined samples.

	Cattle		Water Buffalo		Ticks *	
Province	*n*	No. (%) of *C. burnetii-*positive	*n*	No. (%) of *C. burnetii-* positive	*n*	No. (%) of *C. burnetii-* positive
Cavite	100	0	0	--	89	0
Laguna	111	0	11	0	18	0
Batangas	120	0	8	0	50	0
Rizal	87	2 (2.3)	0	--	0	--
Quezon	94	5 (5.3)	89	3 (3.4)	49	5 (10.2)
Total	512	7 (1.4)	108	3 (2.8)	206	5 (2.4)

* as pooled samples.

## Supplementary Materials

The following are available online at https://www.mdpi.com/2414-6366/5/2/54/s1. Table S1: Primers used for the amplification of control genes *actin* and *mt-rrs* in blood and tick samples, respectively, and detection of *Rickettsia* and *Coxiella burnetii*; Table S2: PCR conditions for the amplification of target fragments of control genes *actin* and *mt-rrs* in blood and tick samples, respectively, and the *gltA* gene of *Rickettsia* and *com1* gene of *C. burnetii*.
