# Supplementary material for: Molecular Detection of Rickettsia spp. and Coxiella burnetii in Cattle, Water Buffalo, and Rhipicephalus (Boophilus) microplus Ticks in Luzon Island of the Philippines
Source: Trop Med Infect Dis. 2020 Apr 4;5(2):54. doi: 10.3390/tropicalmed5020054 (PMC7345074; doi:10.3390/tropicalmed5020054)
Supplement: Supplementary file 1 [file tropicalmed-05-00054-s001.pdf]

Tropical Medicine and Infectious Disease

MDPI

Article(supplementary)

Molecular Detection of *Rickettsia* Spp. and *Coxiella Burnetii* in Cattle, Water Buffalo, and *Rhipicephalus* (*Boophilus*) *Microplus* Ticks in Luzon Island of the Philippines

Remil L. Galay [1,*], Melbourne R. Talactac [2], Bea V. Ambita-Salem [1], Dawn Maureen M. Chu [1], Lali Marie O. dela Costa [1], Cinnamon Mae A. Salangsang [1], Darwin Kyle B. Caracas [1], Florante H. Generoso [2], Jonathan A. Babelonia [2], Joeneil L. Vergano [2], Lena C. Berana [2], Kristina Andrea C. Sandalo [1], Billy P. Divina [1], Cherry R. Alvarez [2], Emmanuel R. Mago [2], Masako Andoh [3] and Tetsuya Tanaka [4,*]

1 Department of Veterinary Paraclinical Sciences, College of Veterinary Medicine, University of the Philippines Los Baños, College, Laguna 4031, Philippines; Beaambita@gmail.com (B.V.A.); dmchu@up.edu.ph (D.M.M.C.); lodelacosta@up.edu.ph (L.M.O.C.); casalangsang@up.edu.ph (C.M.A.S.); dbcaracas@up.edu.ph (D.K.B.C.); kacsandalo@gmail.com (K.A.C.S.); bpdivina@up.edu.ph (B.P.D.)

2 Department of Clinical and Population Health, College of Veterinary Medicine and Biomedical Sciences, Cavite State University, Indang, Cavite 4122, Philippines; melbourne.talactac@gmail.com (M.R.T.); florantegeneroso@gmail.com (F.H.G.); jonathanbabelonia@gmail.com (J.A.B.); joeneilbergs@gmail.com (J.L.V.); lenaberana777@gmail.com (L.C.B.); cherryreyesalvarez@gmail.com (C.R.A.); emman.mago.7081@gmail.com (E.R.M.)

3 Laboratory of Public Health, Joint Faculty of Veterinary Medicine, Kagoshima University, 1-21-24 Korimoto, Kagoshima 890-0065, Japan; masako@vet.kagoshima-u.ac.jp

4 Laboratory of Infectious Diseases, Joint Faculty of Veterinary Medicine, Kagoshima University, 1-21-24 Korimoto, Kagoshima 890-0065, Japan

* Correspondence: rlgalay@up.edu.ph (R.L.G.); k6199431@kadai.jp (T.T.); Tel.: +63-049-536-2728 (R.L.G.); Tel: +81-99-285-3570 (T.T.)

Received: 10 March 2020; Accepted: 02 April 2020; Published: date

Table S1. Primers used for the amplification of control genes *actin* and *mt-rrs* in blood and tick samples, respectively, and detection of *Rickettsia* and *Coxiella burnetii*.

Organism	Target gene	Primer Name	Sequence (5' → 3')	Expected size (bp)	References
Ruminant (Blood)	*actin*	*actin* forward	CGCACCACCGGCATCG TGAT	227	[35]
		actin reverse	TCCAGGGCCACGTAGC AGAG		
Ticks	*mt-rrs*	*mt-rrs* forward	CTGCTCAATGATTTTT AAATTGCTGTGG	560	[36]
		mt-rrs forward	CCGGTCTGAACTCAGA TCAAGTA		
Rickettsia	*gltA*	CS2d	ATGACCAATGAAAATA ATAAT	1,250	[37]
		CSEndr	CTTATACTCTCTATGTA CA		
		RpCs877p	GGGGGCCTGCTCACGG CGG	341	

		RpCs1258n	ATTGCAAAAAGTACAG TGAAC		
Coxiella burnetti	*com1*	OMP1	AGTAGAAGCATCCCAA GCATTG	501	[38]
		OMP2	TGCCTGCTAGCTGTAA CGATTG		
		OMP3	GAAGCGCAACAAGAA GAACAC	438	
		OMP4	TTGGAAGTTATCACGC AGTTG		

Table S2. PCR conditions for the amplification of target fragments of control genes *actin* and *mt-rrs* in blood and tick samples, respectively, and the *gltA* gene of *Rickettsia* and *com1* gene of *C. burnetti*.

Target Gene	PCR Condition
actin	$\frac{94^{\circ}C}{5\ min} \rightarrow [\frac{94^{\circ}C}{30\ s} - \frac{65^{\circ}C}{30\ s} - \frac{72^{\circ}C}{30\ s}]\ 35x \rightarrow \frac{72^{\circ}C}{5\ min} \rightarrow 10^{\circ}C\ \infty$
mt-rrs	$\frac{95^{\circ}C}{5\ min} \rightarrow [\frac{94^{\circ}C}{30\ s} - \frac{56^{\circ}C}{30\ s} - \frac{72^{\circ}C}{30\ s}]\ 30x \rightarrow \frac{72^{\circ}C}{5\ min} \rightarrow 10^{\circ}C\ \infty$
gltA	1st round: $\frac{95^{\circ}C}{10\ min} \rightarrow [\frac{94^{\circ}C}{30\ s} - \frac{52^{\circ}C}{30\ s} - \frac{72^{\circ}C}{1.5\ min}]\ 30x \rightarrow \frac{72^{\circ}C}{5\ min} \rightarrow 10^{\circ}C\ \infty$
	2nd round: $\frac{95^{\circ}C}{10\ min} \rightarrow [\frac{94^{\circ}C}{30\ s} - \frac{52^{\circ}C}{30\ s} - \frac{72^{\circ}C}{30\ s}]\ 30x \rightarrow \frac{72^{\circ}C}{5\ min} \rightarrow 10^{\circ}C\ \infty$
com1	1st round: $\frac{94^{\circ}C}{5\ min} \rightarrow [\frac{94^{\circ}C}{1\ min} - \frac{54^{\circ}C}{1\ min} - \frac{72^{\circ}C}{1\ min}]\ 36x \rightarrow \frac{72^{\circ}C}{5\ min} \rightarrow 10^{\circ}C\ \infty$
	2nd round: $\frac{94^{\circ}C}{3\ min} \rightarrow [\frac{94^{\circ}C}{30\ s} - \frac{54^{\circ}C}{1\ min} - \frac{72^{\circ}C}{30\ s}]\ 36x \rightarrow \frac{72^{\circ}C}{5\ min} \rightarrow 10^{\circ}C\ \infty$
